# Early prediction of plastic bronchitis in pediatric patients with *Mycoplasma pneumoniae* pneumonia by interpretable machine learning algorithms

**DOI:** 10.3389/fcimb.2026.1785189

**Published:** 2026-04-23

**Authors:** Pei Wang, Rui Duan, Qiong Wang, Di Xiao

**Affiliations:** 1 Department of Laboratory Medicine, Jingmen Central Hospital, Jingmen, China; 2 Department of Medicine, Jingmen Central Hospital Affiliated with Jingchu University of Technology, Jingmen, China; 3 Department of Pediatrics, Jingmen Central Hospital, Jingmen, China; 4 Department of Comprehensive Maternal and Child Health, Guangzhou Women and Children’s Medical Center, Guangzhou Medical University, Guangdong Provincial Clinical Research Center for Child Health, Guangzhou, China

**Keywords:** bronchoscopy, machine learning, mycoplasma pneumoniae pneumonia, plastic bronchitis, predictive models

## Abstract

**Background:**

*Mycoplasma pneumoniae* pneumonia (MPP) can cause plastic bronchitis (PB), a rare, life-threatening condition. However, current diagnostic methods often fail to identify early-stage PB in children.The aim of our study was to develop machine learning algorithms to identify early-stage PB in pediatric patients with MPP.

**Methods:**

This retrospective cohort study involved 307 pediatric patients with MPP who underwent bronchoscopy intervention from April 2023, to June 2025.Patients were randomly split into training and test sets (7:3). After feature selection using LASSO and Boruta algorithms, four algorithms, namely, extreme gradient boosting (XGBoost), logistic regression, random forest, and support vector machine, were employed to construct machine learning (ML) models through 5-fold cross-validation. Model performance was evaluated using the area under the curve (AUC), calibration curves, and decision curve analysis (DCA). The best-performing ML was selected using AUC, and feature importance in the model was ranked using SHapley Additive exPlanations (SHAP). Finally, a web-based risk predictor was constructed to facilitate user operability.

**Results:**

MPP children with PB demonstrated more significant abnormalities in inflammation- and nutrition-related indices compared to those without PB. The XGBoost algorithm exhibited the best predictive performance, surpassing other models (logistic regression, random forest, and support vector machine) with an AUC of 0.948 (95% CI: 0.919–0.973), a sensitivity of 0.904, and a specificity of 0.858 on the training set, and an AUC of 0.905 (95% CI: 0.843–0.957), a sensitivity of 0.812, and a specificity of 0.852 on the test set. This algorithm also presented good calibration and net clinical benefit. SHAP analysis identified the retinol-binding protein 4 level, M. pneumoniae cycle-threshold value, D-dimer level, fever duration before admission, C-reactive protein-to-albumin ratio, and presence of pleural effusion as key predictors. To facilitate the clinical adoption, a freely accessible online calculator has been developed (https://plasticbronchitis.shinyapps.io/plastic_bronchitis_risk_calculator/).

**Conclusion:**

The developed interpretable ML models deployed in the network application can help clinicians identify children at high risk of developing PB earlier and tailor timely bronchoscopy intervention and nutritional support as well as anti-inflammatory therapy.

## 1 Introduction

Mycoplasma pneumoniae (MP) is among the primary pathogens that causes community-acquired pneumonia (CAP) in children (Jain et al., 2015; Kutty et al., 2019). MP infection can lead to severe CAP that requires hospitalization and may be accompanied by extrapulmonary complications, such as plastic bronchitis (PB), a pulmonary disease characterized by the formation of thick mucus plugs within the airways leading to severe respiratory obstruction (Kallam et al., 2021). PB occurs more frequently in children with MP pneumonia (MPP) than in children with community-acquired pneumonia caused by other pathogens (Jiang et al., 2026). Since April 2023, a significant resurgence in MP infections has been observed in China and other countries (Meyer Sauteur and Beeton, 2024), accompanied by a relatively high proportion of severe MPP (SMPP) and macrolide-resistant MPP (MRMP) cases (Méndez-Echevarría et al., 2024; Bolormaa et al., 2025).

Unfortunately, both SMPP and MRMP are independent risk factors for PB formation in children (Wang et al., 2025a; Chunlian et al., 2025). Although fiberoptic bronchoscopy (FOB) is the gold standard for the diagnosis and treatment of PB (Kallam et al., 2021), early recognition of PB remains challenging because its symptoms and clinical presentations overlap with those of similar conditions, which may cause misdiagnosis and missed diagnosis (Ntiamoah et al., 2021; Wang et al., 2025b). For example, distinguishing refractory MPP from PB based on symptoms, signs and imaging findings is difficult (Zhang et al., 2023a). More importantly, bronchoscopy is not suitable for routine screening of PB in children due to its invasiveness. Therefore, evaluating PB risk and facilitating early intervention in MPP children is an urgent need. Numerous studies have been focused on investigating the risk factors for PB, primarily by employing conventional linear logistic regression (LR) approaches (Wang et al., 2025a; Zhang et al., 2023a; Zhao et al., 2022; Yang et al., 2023; Jiang et al., 2025; Huang et al., 2025; Lin et al., 2024). Conventional statistical approaches, while valuable, often fall short in handling the complexity of high-dimensional data (D’Ascenzo et al., 2021). Currently, convenient and accurate models to quantify these risk factors and assess PB risk at the early stage in MPP patients are still lacking.

The advent of powerful machine learning (ML) algorithms has added new fuel to the diagnosis of infectious diseases (Xu et al., 2025a), opening new unprecedented options for deriving highly predictive models for assisting in the diagnosis of pediatric MPP and SMPP (Gong et al., 2025; Xie et al., 2025; Guo and Luo, 2025; Liu et al., 2025). To date, however, only a few studies have explored the efficacy of ML models for predicting PB (Li et al., 2025; Xu et al., 2025b). Moreover, these studies either used a decision tree model or multifactorial model for their predictions, both of which lack the interpretability required to fully decipher the complex relationships among variables (Chen et al., 2024). To address these issues, the development of new algorithms is essential to enhance early prediction of PB. Therefore, four predictive models, namely, extreme gradient boosting (XGBoost), LR, random forest (RF), and support vector machine (SVM), were leveraged to investigate the risk factors associated with PB in children with MPP and provide early prediction and timely intervention. In this study, our aims were to assess the effectiveness of ML algorithms in the early identification of pediatric PB and provide an accessible web-based tool for clinicians to manage pediatric patients with MPP.

## 2 Materials and methods

### 2.1 Study population and design

**Figure 1:**
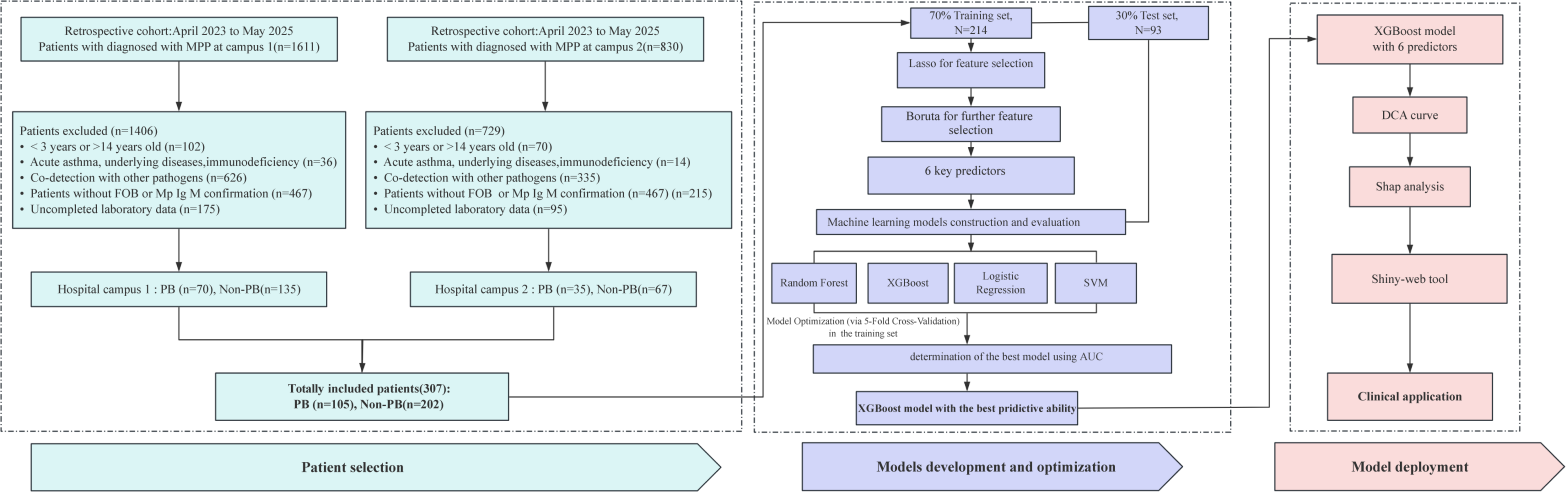
Flow chart of the study design. FOB, fiber optic bronchoscopy; LASSO, least absolute shrinkage and selection operator; RF, random forest; XGBoost, extreme gradient boosting; LR, logistic regression; SVM, support vector machine; AUC, area under the curve; DCA, decision curve analysis; SHAP, SHapley additive explanation.

The research design for this study is illustrated in **Figure 1**.This retrospective study was conducted at Jingmen Central Hospital from April 2023 to June 2025 at two hospital campuses. A total of 205 patients were included from the campus 1, while 102 patients were included from campus 2. Both cohorts adhered to the same inclusion and exclusion criteria. All patients were randomly split into a training set (70%; 214) and a test set (30%; 93). Eligible participants were those who had a positive MP DNA polymerase chain reaction test according to nasopharyngeal swab or bronchoalveolar lavage fluid (BALF) samples and a positive serological test for MP immunoglobulin M (IgM), underwent fiberoptic bronchoscopy, were aged between 3 and 14 years at the time of recruitment, presented fever and respiratory symptoms, and had pneumonia confirmed by a radiological diagnosis. Patients were excluded if they had coinfections with other pathogens (determined by targeted next-generation sequencing from BALF samples), acute asthma, underlying diseases, immunodeficiency, tuberculosis, chronic malnutrition, or insufficient data, such as missing bronchoscopy examinations or laboratory tests.

### 2.2 Sample size calculation

The rule of events per variable criterion (EPV) ≥10 was utilized to estimate the minimal required sample size (Peduzzi et al., 1996). Given our intention to include six predictor variables, the incidence of PB was 0.34 in the training cohort, the required sample size for the training cohort via the following formula (Yuan et al., 2025): sample size=(number of variables× EPV)/incidence rate=(6×10)/0.34 = 177.

Assuming the training to test cohort ration (7:3), the minimum required sample size was calculated to be 253. To improve the generalizability of the results, we further accounted for a 10% invalid sample, resulting in a final minimum sample size of 278.

### 2.3 Definition of study outcomes

The diagnosis of SMPP adhered to Chinese guidelines (Expert Committee on Rational Use of Medicines for Children Pharmaceutical Group, National Health and Family Planning Commission, 2020), which in turn are in accordance with the WHO definition. The diagnosis of PB was made as described in our previous study: PB was confirmed when solidified mucus plugs or casts shaped like the bronchial tree were removed by fiber optic bronchoscopy (Wang et al., 2025a). Patients were divided into a PB group and a non-PB group according to the bronchoscopy findings. Bronchoscopy was performed when an MP infection with solid consolidation or pulmonary atelectasis did not improve, there was a poor response to treatment, or the diagnostic criteria for SMPP were fulfilled (Wang et al., 2025a).

### 2.4 Data acquisition and analysis

Demographic characteristics, clinical and laboratory data, and radiographic findings were retrieved from the patients’ hospitalization electronic medical records (EMRs). Specifically, the following data were extracted: (1) Clinical characteristics: Age, sex, symptom duration, fever duration before admission, maximum body temperature (BT), length of hospital stay (LOS), and prehospital antibiotic use. (2) FOB findings. (3) Radiographic findings: presence of pulmonary consolidation, pleural effusion, or pericardial effusion. (4) Laboratory parameters: the Complete blood count (white blood cells, absolute neutrophils, lymphocytes, and platelets); biochemical markers (lactate dehydrogenase [LDH], prealbumin [PA], albumin [ALB], retinol-binding protein 4 [RBP4], and serum amyloid A [SAA]); and coagulation markers (D-dimer [DD]). Microbiological results, including the MP DNA Ct values and MRMP status, were also recorded. Hematological indices—the neutrophil-to-lymphocyte ratio (NLR), platelet-to-lymphocyte ratio (PLR), CRP-to-albumin ratio (CAR) and systemic immune-inflammation index (SII, calculated as (platelet count × neutrophil count)/lymphocyte count)—were derived. All laboratory tests were performed within 24 hours of admission. All assay methods are provided in **Supplementary Table 1**. No missing data were observed for any study variables, and therefore no imputation was performed.

### 2.5 Machine learning model development and validation

For the purpose of creating and validating the models, the data set was randomly partitioned into a training set (70%) and a test set (30%) using stratified sampling to maintain the outcome distribution. The training set was used for training and optimization of the models, and the test set was used to evaluate and compare the performance of the models. To prevent data leakage, all the preprocessing steps—including one-hot encoding for categorical variables and Z score normalization for continuous variables—were performed on the training set and subsequently applied to the test set.

Least absolute shrinkage and selection operator (LASSO) regression is an improved method used for feature selection, which achieves feature selection by penalizing coefficients to prevent overfitting; the Boruta algorithm is based on random forest for feature selection, and the selection frequency is based on multiple iterations of the feature selection procedure. In addition, these two methods are suitable for a dataset with a small sample size. Therefore, feature selection was performed using a two-stage approach. Initially, least absolute shrinkage and selection operator (LASSO) regression with tenfold cross-validation was used to identify features with nonzero coefficients. The Boruta algorithm was subsequently applied, and variables marked as “Confirmed” or “Tentative” were selected for final model construction.

Four machine learning algorithms were used to construct the models: XGBoost, LR, RF, and SVM. Model performance was evaluated using stratified 5-fold cross-validation with AUC as the performance metric. The exact parameter configurations for each model are provided in **Supplementary Table 2**.

### 2.6 Statistical analysis

Continuous variables are presented as the mean ± standard deviation or median (interquartile range [IQR]) and were compared using Student’s t test or the Mann–Whitney U test, as appropriate. Categorical variables are presented as counts (percentages) and were compared using the chi-square test or Fisher’s exact test.

Model discrimination was assessed using the AUC with its 95% confidence interval (CI), calculated via bootstrap resampling (1, 000 iterations). AUC is a performance indicator used for assessment of the quality of a classifier, and a higher AUC value reflects better performance of the classifier. Discrimination performance was classified according to the AUC as moderate (0.70–0.80), good (0.80–0.90), or excellent (>0.90) (Muller et al., 2005). Additional metrics included accuracy, sensitivity, specificity, the positive predictive value (PPV), the negative predictive value (NPV), the F1 score, and the Brier score. Model calibration was evaluated with calibration curve analysis and the Hosmer–Lemeshow test. Decision curve analysis was performed to assess the clinical utility of the models. The optimal binary classification threshold was determined using the Youden index, which maximizes the sum of sensitivity and specificity on the training set. Patients with a predicted probability at or above this threshold were classified as high risk, and those with a predicted probability below the threshold were classified as low risk. Finally, feature importance was quantified using SHapley Additive exPlanations (SHAP).

All analyses were performed using Python (version 3.13.5) and SPSS (version 27.0, Armonk, NY, USA). A two-sided *P* value < 0.05 was considered to indicate statistical significance.

### 2.7 Web-based risk calculator

To facilitate model accessibility in a clinical setting, the best-performing model was integrated into a Shiny-based web platform as an application. The user can input the values of the relevant variables into the application, which then returns the probability that the pediatric MPP patient has PB, along with a force plot for individual features.

## 3 Results

### 3.1 Demographic and clinical characteristics

The data of 307 patients hospitalized from April 2023 to June 2025 were included in this study. Patient characteristics, as presented in **Table 1**, were analyzed before modeling to compare the non-PB group (*n* = 202) with the PB group (*n* = 105). The median age was 9.0 years (IQR = 6.25–11.0) in the non-PB group and 9.0 years (IQR = 7.0–11.0) for the PB group. A total of 46.5% of the non-PB group and 45.7% of the PB group were male. No significant differences were observed between the two groups in terms of age or sex distribution. According to the univariable analysis, the maximum BT, fever duration before admission, LOS, the proportion of patients with SMPP, pulmonary consolidation, pericardial effusion, and pleural effusion, the MP Ct value, RBP4 level, CRP level, CAR, ALB level, SAA level, DD level and absolute neutrophil count (ANC) were significantly associated with PB (*P* < 0.05), whereas the remaining variables were not.

**Table 1:** Clinical and laboratory characteristics of the study patients.

Characteristic	Total (n=307)	Non-PB group (n=202)	PB group (n=105)	*P* value
Age, years (IQR)	9.00 (7.00, 11.00)	9.00 (6.25, 11.00)	9.00 (7.00, 11.00)	0.942
Sex, male	142 (46.3%)	94 (46.5%)	48 (45.7%)	0.891
PS duration, days (IQR)	7.00 (5.00, 10.00)	7.00 (4.25, 9.75)	8.00 (5.00, 11.00)	0.161
Fever duration, days (IQR)	4.00 (3.00, 6.00)	4.00 (3.00, 5.00)	5.00 (4.00, 7.00)	<0.001
Maximum BT, °C (IQR)	39.00 (38.70, 39.70)	39.00 (38.70, 39.60)	39.10 (38.80, 40.00)	0.024
MRMP	193 (62.9%)	122 (60.4%)	71(67.6%)	0.214
Antibiotic self-medication	192 (62.5%)	120 (59.4%)	72 (68.6%)	0.115
LOS, days (IQR)	8.00 (6.00, 9.00)	7.00 (6.00, 9.00)	8.00 (7.00, 9.00)	0.003
Pulmonary consolidation	208 (67.8%)	116 (57.4%)	92 (87.6%)	<0.001
Pericardial effusion	8 (2.6%)	2 (1.0%)	6 (5.7%)	0.021
Pleural effusion	63 (20.5%)	18 (8.9%)	45 (42.9%)	<0.001
Severe MPP	72 (23.5%)	29 (14.4%)	43 (41.0%)	< 0.001
WBC, × 10^9^/L	6.20 (4.90, 7.40)	5.90 (4.80, 7.20)	6.30 (5.20, 7.60)	0.070
LYM count, × 10^9^/L	1.60 (1.30, 2.00)	1.60 (1.30, 2.00)	1.60 (1.30, 2.00)	0.929
ANC × 10^9^/L	3.70 (2.80, 4.80)	3.50 (2.70, 4.67)	3.90 (3.00, 5.10)	0.032
PLT count, × 10^9^/L	238.00 (208.00, 292.00)	238.00 (209.00, 289.50)	239.00 (206.00, 292.00)	0.896
LDH, U/L	243.00 (212.80, 280.50)	242.50 (211.25, 274.00)	249.00 (213.00, 299.00)	0.090
PA, mg/L	116.00 (100.30, 150.75)	117.00 (104.00, 155.00)	110.00 (94.70, 140.00)	0.052
ALB, g/L	43.00 (41.00, 45.00)	43.00 (41.00, 45.00)	42.20 (39.60, 44.60)	0.042
CRP, mg/L	8.60 (3.60, 18.90)	5.75 (3.10, 15.88)	15.30 (7.00, 25.00)	< 0.001
CAR	0.21 (0.08, 0.45)	0.14 (0.07, 0.37)	0.36 (0.16, 0.60)	< 0.001
SAA, mg/L	78.00 (37.10, 243.00)	65.00 (31.00, 203.95)	150.80 (42.00, 376.00)	< 0.001
RBP4, mg/L	19.00 (14.40, 24.00)	21.45(17.92, 26.00)	14.40 (11.40, 17.40)	< 0.001
DD, µg/ml FEU	0.60 (0.50, 0.90)	0.50 (0.40, 0.80)	0.80 (0.60, 1.20)	< 0.001
NLR	2.30 (1.70, 3.10)	2.30 (1.60, 3.10)	2.44 (1.79, 3.45)	0.101
PLR	149.09 (117.09, 188.26)	152.59 (120.84, 186.35)	142.86 (112.38, 191.67)	0.444
SII	554.12 (371.74, 786.05)	543.21(371.55, 742.40)	583.41 (385.42, 858.00)	0.337
Ct values	28.00 (25.00, 32.00)	30.00 (27.00, 34.00)	25.00 (23.00, 28.00)	< 0.001

PS duration, prodromal symptom duration; maximum BT, maximum body temperature; MRMP, macrolide-resistant *Mycoplasma pneumoniae*; LOS, length of hospital stay; MPP, *Mycoplasma pneumoniae* pneumonia; WBC, white blood cell; LYM, lymphocyte; ANC, absolute neutrophil count; PLT, platelet; LDH, lactate dehydrogenase; PA, prealbumin; ALB, albumin; CRP, C-reactive protein; CAR, CRP-to-albumin ratio; SAA, serum amyloid protein A; RBP, retinol-binding protein; DD, D-dimer; NLR, neutrophil-to-lymphocyte ratio; PLR, platelet-to-lymphocyte ratio; SII, systemic inflammation response index; Ct values, cycle threshold values.

Continuous variables were compared using the Mann–Whitney U test. Categorical variables were compared using the chi-square test or Fisher’s exact test.

### 3.2 Feature selection

**Figure 2:**
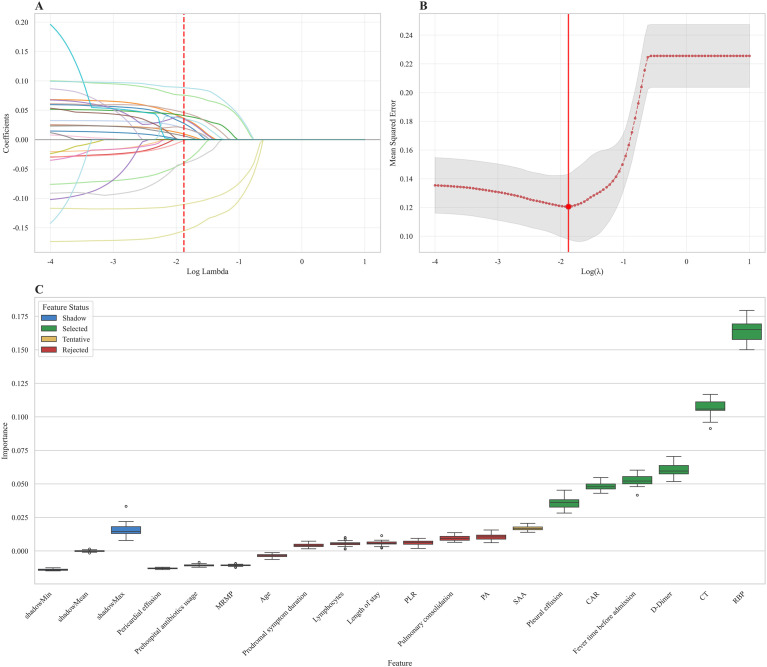
LASSO and Boruta algorithms. LASSO regression coefficient plot **(A)**. LASSO regression cross-validation curve **(B)**. The importance of shadow and predictor variables selected by the Boruta algorithm **(C)**. LASSO, least absolute shrinkage and selection operator.

We subsequently used LASSO regression and the Boruta method to identify relevant features in the training set. First, we performed LASSO regression analysis (with an optimal lambda of 0.013) to screen for predictors of PB; ultimately, the following variables were retained: pericardial effusion, prehospital antibiotic usage, the presence of MRMP, age, prodromal symptom duration, lymphocyte count, length of stay, the PLR, the presence of pulmonary consolidation, PA level, SAA level, the presence of pleural effusion, CAR, fever duration before admission, DD level, MP Ct value, and RBP4 level (**Figures 2A, B**); the regression coefficients for the variables in the LASSO regression are provided in **Supplementary Table 3**. Next, the Boruta algorithm was applied to filter irrelevant and redundant features on the basis of their Z values. The RBP4 level, MP Ct value, DD level, fever duration before admission, CAR and the presence of pleural effusion were identified as confirmed variables (**Figure 2C**). Finally, a feature set consisting of these variables was used for model development and evaluation.

### 3.3 Model performance comparisons

**Figure 3:**
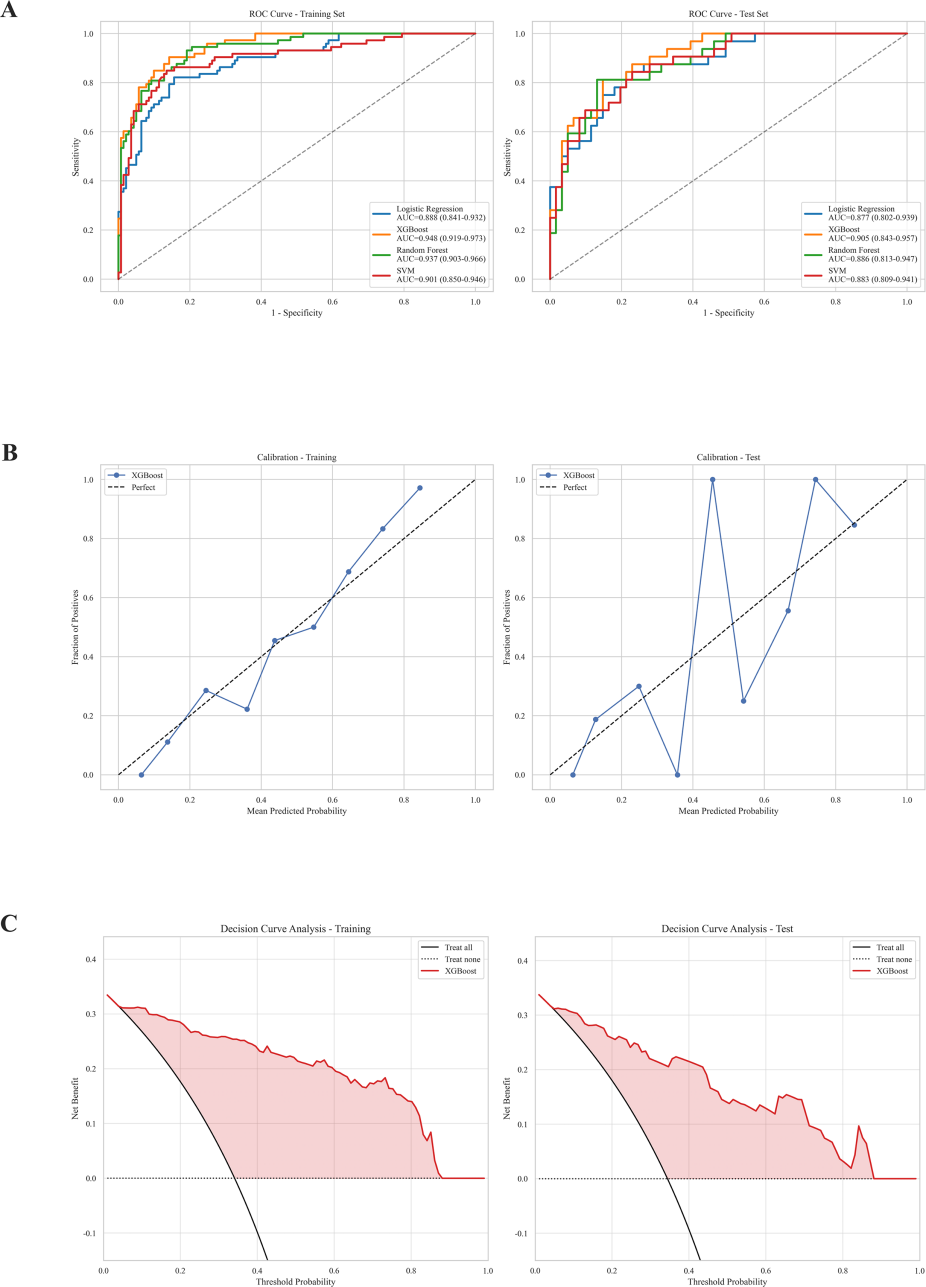
**(A)** ROC curves of the four prediction models in the training set (left), and the test set (right). **(B)** calibration curves of the XGBoost model in the training set(left), and the test set (right). **(C)** DCA plot of the XGBoost model in the training set(left), and the test set (right). LASSO, least absolute shrinkage and selection operator; RF, random forest; XGBoost, extreme gradient boosting; LR, logistic regression; SVM, support vector machine.

The six selected predictors were used to train and evaluate the four ML models, while 5-fold cross-validation was conducted during training to determine the hyperparameters for each model. The performance of the four machine learning models (LR, XGBoost, RF, and SVM) was compared according to the AUC, accuracy, sensitivity specificity, PPV, NPV, and F1 score in both the training and test sets (**Table 2**, **Figure 3A**).

**Table 2:** Performance comparison of the four machine learning models.

Model	AUC (95% CI)	Accuracy	Sensitivity	Specificity	PPV	NPV	F1 score
Training set							
LR	0.888 (0.841-0.932)	0.836	0.822	0.844	0.732	0.902	0.774
XGBoost	0.948 (0.919-0.973)	0.874	0.904	0.858	0.767	0.945	0.830
RF	0.937 (0.903-0.966)	0.850	0.932	0.809	0.716	0.958	0.810
SVM	0.901 (0.850-0.946)	0.790	0.904	0.730	0.635	0.936	0.746
Test set							
LR	0.877 (0.802-0.939)	0.785	0.781	0.787	0.658	0.873	0.714
XGBoost	0.905 (0.843-0.957)	0.839	0.812	0.852	0.743	0.897	0.776
RF	0.886 (0.813-0.947)	0.774	0.812	0.754	0.634	0.885	0.712
SVM	0.883 (0.809-0.941)	0.774	0.844	0.738	0.628	0.900	0.720

LR, logistic regression; XGBoost, extreme gradient boosting; RF, random forest; SVM, support vector machine; AUC, area under the curve; PPV, positive predictive value; NPV, negative predictive value.

In the training set, the XGBoost model demonstrated strong performance (AUC 0.948, 95% CI: 0.919–0.973; accuracy 0.874; sensitivity 0.904; specificity 0.858; PPV 0.767; NPV 0.945; and F1 score 0.830) and outperformed the LR (AUC 0.888), RF (AUC 0.937) and SVM (AUC 0.901) models (**Table 2**, **Figure 3A**).

When evaluated in the test set, XGBoost performed well across most key metrics (**Table 2**, **Figure 3A**), achieving an AUC of 0.905 (95% CI: 0.843–0.957), an accuracy of 0.839, a sensitivity of 0.812, a specificity of 0.852, a PPV of 0.743, an NPV of 0.897, and an F1 score of 0.776. The Hosmer–Lemeshow test confirmed an adequate fit and good model calibration in both the training (χ^2^ = 13.75, *P* = 0.089) and test (χ^2^ = 6.36, *P* = 0.606) sets, while the corresponding Brier scores were 0.091 and 0.124, respectively (**Figure 3B**). The Brier score combines the differentiation and calibration of the model and is utilized to assess the overall performance of the model. The closer to zero the Brier score is, the smaller the forecast error and the better the prediction supporting model reliability. Decision curve analysis revealed that the XGBoost model provided a net positive benefit across a broad range of threshold values (**Figure 3C**), suggesting its potential applicability in clinical practice. Given its overall advantages, the XGBoost model was selected as the optimal algorithm. This model was subsequently utilized for interpretability analyses and the development of a web-based predictive tool. The Youden-index-derived optimal threshold was 0.374, which was used to classify patients into high-risk and low-risk groups.

### 3.4 Interpretability analysis

**Figure 4:**
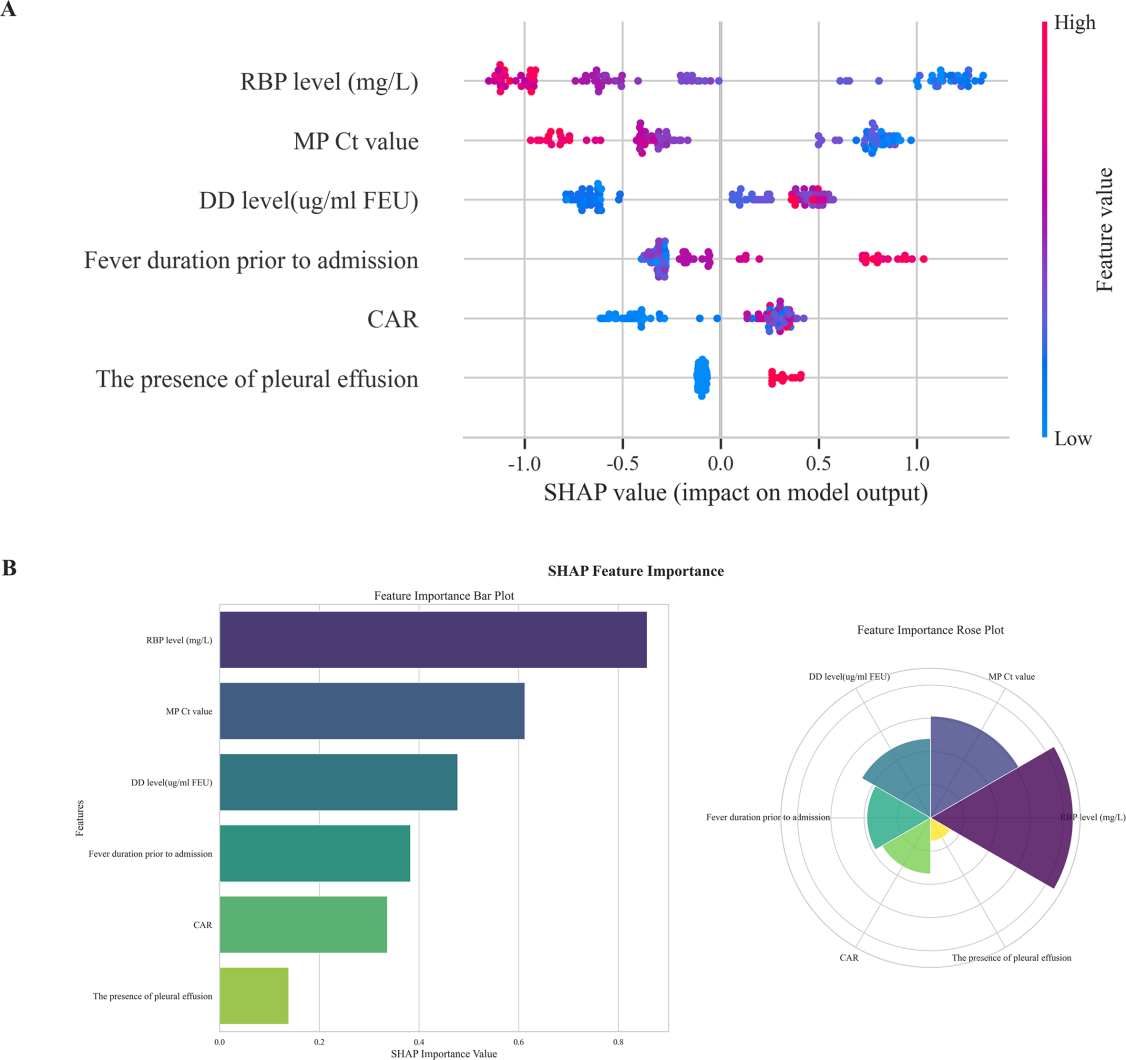
Model feature importance explanation using the SHAP method. SHAP summary dot plot **(A)**. Each dot represents the SHAP value of a model feature for a patient. The *x* axis represents the SHAP value, and the color from red to blue represents high to low feature values, respectively. SHAP feature importance bar and rose plots **(B)**. The features are visually ranked by their average impact on model predictions using SHAP values. SHAP, Shapley additive explanation; RBP, Retinol binding protein; Ct value, Cycle threshold value; DD, D-dimer; CAR, C-reactive protein-to-albumin ratio; PE, Pleural effusion.

SHAP values were calculated to determine the importance of the features in the XGBoost model. The SHAP summary dot plot (**Figure 4A**) illustrates the direction and strength of the influence of each feature on the model’s predictions. In addition, the SHAP summary bar plot (**Figure 4B**) illustrates the contribution of the features to the model, as indicated by the mean SHAP values, which are displayed in the following descending order: RBP4 level, Ct value from polymerase chain reaction, DD level, fever duration before admission, C-reactive protein-to-albumin ratio, and the presence of pleural effusion.

### 3.5 Development of a web-based risk calculator

**Figure 5:**
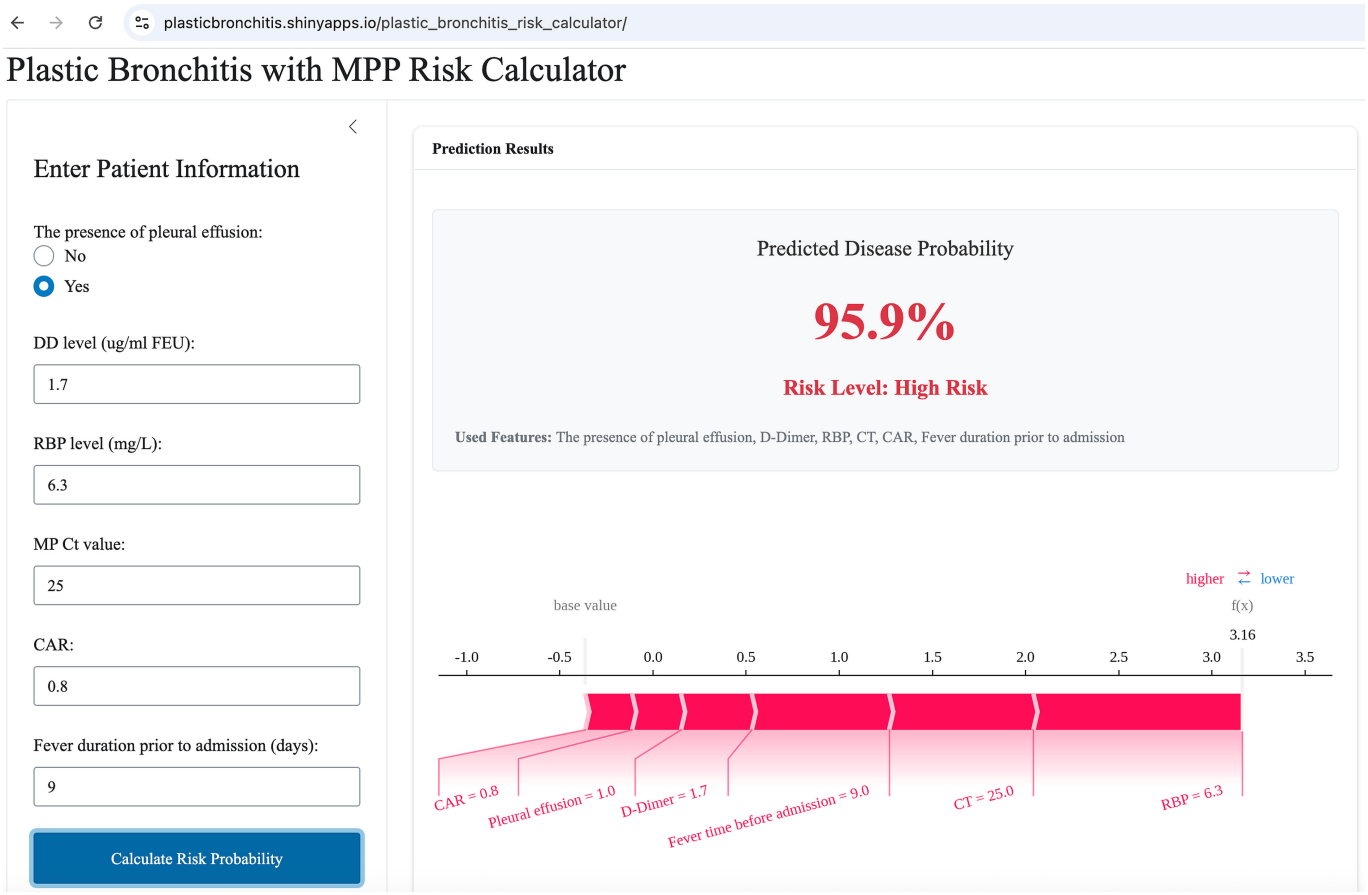
Screenshot of the interface of the web-based tool for plastic bronchitis (PB) risk prediction. Additionally, a force plot can be displayed simultaneously depicting the influence of each feature on the prediction outcome.

The developed prediction model is accessible on the internet via Shiny, online at (https://plasticbronchitis.shinyapps.io/plastic_bronchitis_risk_calculator/). The application allows a pediatrician to enter the six predictive variables and instantly obtain an individualized predicted probability for PB and the corresponding risk classification (high risk or low risk). Using the selected patient as an example (**Figure 5**), the predicted probability was 95.9%, and the patient was classified as high risk. SHAP force plots provide an intuitive visualization of how different features affect an individual prediction. Red features on the left increase the likelihood of PB, whereas blue features on the right decrease it, clearly delineating the factors that drive the prediction of the model (**Figure 5**).

## 4 Discussion

In this study, we proposed four ML models to forecast the risks of developing PB in pediatric patients with MPP using data obtained through electronic medical records. The XGBoost model outperformed the LR, RF, SVM models and achieved the best performance. This approach has solved the problem of distinguishing PB from pneumonia in children prior to bronchoscopy (Cai et al., 2025), and the predictive results are available within 2 days upon admission. Importantly, these algorithms are noninvasive, allowing for routine screening of PB without potential risks for children. A web-based risk calculator was developed to offer clinicians a practical tool for the early identification of high-risk patients, thereby assisting in prompt treatment strategies. Regarding its application in clinical practice, if the risk of developing PB predicted by the ML algorithm in a patient is low, the clinician may advise caution and monitor the patient closely, as the possibility of the later development of PB cannot be ruled out; conversely, if the risk prediction shows a high likelihood of PB development, prompt bronchoscopy may be warranted to improve clinical outcomes.

How to define high-risk patients is the key to translate ML model outputs into real-world practice (Fan et al., 2025). Previous studies have also investigated ML-based MPP-related PB prediction. Li et al. explored the predictive value of a decision tree model in diagnosis of MPP with PB and reported a good sensitivity of 0.884 and a moderate specificity of 0.727 (Li et al., 2025). Xu et al. established a ML model integrating radiomics features and pleural effusion to predict PB in children with MPP (Xu et al., 2025b), obtaining a sensitivity of 0.720 and a specificity of 0.760 on the test set. Compared with these two studies, our XGBoost algorithm achieved a more balanced performance in the test set, with a sensitivity of 0.812 and a specificity of 0.852. The performances of these algorithms are inferior to that of our study. The moderate performance and without the interpretability of these models may hamper their clinical use as a predicative tool for PB. Moderate sensitivity and specificity might be unacceptable in clinical settings with high-risk populations because many false positives and negatives are inevitable. A high false positive rate may lead to unnecessary bronchoscopy procedures and potential risks of trauma to the pediatric airway and postoperative complications, whereas a high negative rate could cause delayed treatment and adverse outcomes. The performance of the XGBoost model in this study is consistent with its performance in earlier studies (Guan et al., 2024; Strutz et al., 2025; Demsash et al., 2025). Given the imbalanced nature of this dataset, in which a minority (34.2%) of the children had PB, models such as those based on the SVM and LR algorithms tended to become biased toward the majority class. With the advantage of resistance to overfitting in datasets with imbalanced feature or ratios, the XGBoost model can better handle imbalanced datasets (Vaid et al., 2021).The XGBoost model in our study retained a good sensitivity without incurring a moderate specificity, which guaranteed its accurate prediction of PB in real-world deployment.

Excessive inflammatory immune reactions are postulated to play major roles in the formation of MPP-induced PB. A notable new finding in this study is that the RBP4 level was the most important predictor of PB. Significantly reduced RBP4 levels were observed in the PB group. In line with our finding, Vollenberg et al. confirmed that reduced RBP4 levels are significantly correlated with elevated inflammatory marker levels (Vollenberg et al., 2022). This study demonstrated that PB is characterized by reduced RBP4 levels; elevated CRP, CAR, SAA, and DD levels; elevated ANC count; and the presence of PE. These findings exhibit a strong inverse association between RBP4 levels and changes in parameters reflecting the inflammatory response in patients with PB. Additionally, reduced RBP4 levels were accompanied by an increased incidence of pulmonary consolidation, pleural effusion and SMPP in the PB group. We believe that RBP4 might reflect the inflammatory response and that its levels correlate with the severity of MPP-induced PB. Similarly, one recent study demonstrated that RBP can be used as a biomarker for valid differentiation of severe MPP from general MPP in children (Xie et al., 2025). In a previous study, RBP4 levels successfully predicted the prognosis of chronic obstructive pulmonary disease (Jin et al., 2013). In light of these findings, RBP4 might be not only an independent predictor of PB but also a promising biomarker of the severity and prognosis of MPP-induced PB.

All abnormalities in clinical and laboratory indicators might be triggered by a higher load of MP infection. Indeed, lower values of the MP Ct value (indicating a greater MP load) were determined in the respiratory specimens and BALF of MPP children with PB. The MP DNA load is correlated with the intensity of the inflammatory response; thus, higher MP infection load makes these patients more likely to experience pronounced immune–inflammation responses (Wang et al., 2024). Even for the DD, a thrombo-inflammatory biomarker, its elevated levels in MPP children also arise from the severe inflammatory process (Kamin Mukaz et al., 2021). Taken together, these results reinforce the importance of excessive inflammation in PB formation in the clinical setting, indicating that anti-inflammatory therapy should be initiated early in PB children to avoid the excessive release of inflammatory mediators leading to disease exacerbation.

Current reports have shown that pleural effusion is a useful imaging index in the prediction of PB. Pleural effusion is a complication of pulmonary infection. Previous studies reported the PE was the most common CT finding in MPP children (Zhang et al., 2023a; Yang et al., 2023; Jiang et al., 2025; Lin et al., 2024).We also observed that the incidence of pleural effusion in the PB group was greater than that in the non-PB group. Increasing pleural effusion and clinical severity after MP infection have been observed since the COVID-19 pandemic (Robinson et al., 2025; Yang et al., 2025), implying a more intensive pulmonary inflammatory response in pediatric MP patients in the post-pandemic era. PB in MPP children often manifests as severe symptoms accompanied by obvious lung imaging changes like PE and pulmonary consolidation (Xu et al., 2025b). Therefore, while being non-specific, presence of PE can serve as an imaging index combined with laboratory and clinical data for predicting the risk of PB in MPP children.

New insights in terms of nutritional–inflammatory biomarkers have emerged from our study; we found the association between PB formation and nutritional–inflammatory biomarkers in MPP children. As the first important predictor for PB, RBP4 is considered as a nutritional–inflammatory biomarker for the escort of vitamin A (Chen et al., 2021). The reduced RBP4 levels in MPP children with PB could indicate Vitamin A deficiency (Zhang et al., 2023b), which could lead to recurrent respiratory infection (Li et al., 2020), such as refractory MPP. Interestingly, another nutritional–inflammatory biomarker CAR was identified as a predictor for PB in this study because CAR reflects both the inflammatory and nutritional status. In addition, CRP increases, acting as a response to infection, trauma, tissue damage, and other inflammatory events, and albumin reflects the nutritional status of a patient and inflammation via a decrease in plasma levels (Eckart et al., 2020). Thus, CAR yields better predictive performance than CRP or albumin alone. Our findings indicate the interplay between nutrition and inflammation in MPP children, which highlights the potential value of timely nutrition support and anti-inflammatory therapy at the early stage of PB to optimize outcomes in MPP patients.

The primary strength of this study is its use of interpretable ML algorithms for the early identification of children with MPP at a high risk of developing PB. Timely bronchoscopic flushing to completely clear the cast results in an improved prognosis. However, achieving this goal is difficult because clinicians often rely solely on imaging to determine whether to perform bronchoscopy on MPP children. PB at the early stage is located in small airways without obvious manifestations on imaging and thus may be overlooked. Atelectasis is a strong indicator for bronchoscopic intervention, but many MPP children with PB present lung consolidation rather than atelectasis on imaging (Ding and Jiang, 2025). ML algorithms have powerful capabilities to detect subtle abnormalities and predict disease progression, allowing MPP children with PB to be differentiated from those without PB before obvious disease manifestations emerge. Furthermore, we developed a real-time, online predictive tool for determining the likelihood of the development of PB in an individual, which translates the model from prediction to practical PB diagnosis and broadens its scope of clinical implementation. Another strength of this study lies in the identification and inclusion of new nutritional–inflammatory predictors, including the serum RBP4 level and CAR, in the predictive models. The integration of novel predictors into ML models not only provides additional diagnostic value but also offers new insights for further exploration of the mechanism of PB formation and provides potential for anti-inflammatory and nutritional support therapy (Liu et al., 2026; Li et al., 2026).

Although our study provides valuable insights into the early and accurate prediction of high-risk children with PB, it also had limitations. First, this study is limited by the lack of external validation on independent datasets, which restricts its robustness and the generalizability of the model and increases the risk of model overfitting. To address this limitation, 5-fold cross-validation was performed to mitigate the risk of overfitting. Although the proposed model demonstrated relatively good performance in internal validation, further external validation using independent datasets is necessary. Second, the study was retrospective in nature, and due to the rarity of PB, the sample size was small, which may have introduced a degree of inherent bias and confounding factors that might have affected our findings. In this regard, a multicenter and large sample size study is needed in the future. Third, since only patients who underwent bronchoscopy were included in this study, the applicability of the model to the entire population of patients with MPP should be approached with caution. Fourth, owing to limited availability of detailed patient histories, some variables such as pre-admission treatment regimens and atelectasis could not be incorporated into the current model, thereby potentially compromising the model’s ability to capture certain predictors. Last, although the best-performing XGBoost model achieved good performance in predicting PB, the sensitivity and specificity can be further improved by incorporating into multiple indicators.

## 5 Conclusion

Machine learning algorithms promote the early identification of children with PB prior to bronchoscopy. The best-performing XGBoost model was further implemented through an online prediction calculator, providing pediatricians with real-time decision-making support and enabling timely bronchoscopy intervention and nutritional support as well as anti-inflammatory therapy.

## Data Availability

The original contributions presented in the study are included in the article/**Supplementary Material**. Further inquiries can be directed to the corresponding author.

## Supplementary material

Supplementary Table 1 Methods of laboratory testing.

Supplementary Table 2 Key parameter configurations for each machine learning model.

Supplementary Table 3 Feature selection with the LASSO algorithm.
