# Supplementary material for: Early prediction of plastic bronchitis in pediatric patients with Mycoplasma pneumoniae pneumonia by interpretable machine learning algorithms
Source: Front Cell Infect Microbiol. 2026 Apr 23;16:1785189. doi: 10.3389/fcimb.2026.1785189 (PMC13149268; doi:10.3389/fcimb.2026.1785189)
Supplement: Supplementary Table 1 — Methods of laboratory testing. [file Table1.docx]

**Table S1. Methods of laboratory testing**

| **Test methods (technique; product)** | **Test parameters** |
| --- | --- |
| PCR (singleplex, real-time; Sansure Biotech Inc. China) | *M. pneumoniae* |
| IgM( YHLO Biotechnology Company, Ltd., Shenzhen, China） | *M. pneumoniae* |
| tNGS (sequencing,KingMed Diagnostics, China) | 153 pathogens (as detailed in S1)*,* MRMP determination |
| Mutations determination (sequencing,KingMed Diagnostics, China) | 23SrRNA A2063G/C/T, C2617G, and/or A2064G/C mutations |
| Hematology analysis (flow cytometry; XN, Sysmex, Kobe, Japan) | WBC, Lymphocytes, ANC, platelet |
| Werfen ACL TOP 750 LAS ( latex-enhanced immunoturbidimetry ,Werfen, MA, USA) | D-Dimer |
| Biochemical analysis (different techniques; Roche Diagnostics, Switzerland) | LDH, ALB, PA, SAA |
| Biochemical analysis (immunoturbidimetric method, Shanghai Beijia Biochemical Reagent Company, Shanghai,China) | RBP4 |

**Abbreviations**: ANC, absolute neutrophil count; LDH, lactate dehydrogenase; MPP, *Mycoplasma pneumoniae* pneumonia; MRMP, macrolide-resistant M. pneumoniae; PA, prealbumin; PCR, polymerase chain reaction; RBP4, retinol-binding protein 4; SAA, serum amyloid A; tNGS, target next-generation sequencing; WBC, white blood cell.
