# Supplementary material for: Early prediction of plastic bronchitis in pediatric patients with Mycoplasma pneumoniae pneumonia by interpretable machine learning algorithms
Source: Front Cell Infect Microbiol. 2026 Apr 23;16:1785189. doi: 10.3389/fcimb.2026.1785189 (PMC13149268; doi:10.3389/fcimb.2026.1785189)
Supplement: Supplementary Table 2 — Key parameter configurations for each machine learning model. [file Table2.docx]

**Supplementary Table 2. Parameter configurations for each machine learning model.**

| **Model** | **Parameter** | **Value** |
| --- | --- | --- |
| Logistic regression | Polynomial degree | 2 |
|  | Solver | lbfgs |
|  | Penalty | L2 |
|  | C (regularization) | 0.002 |
|  | max_iter | 5000 |
| XGBoost | n_estimators | 140 |
|  | max_depth | 3 |
|  | learning_rate | 0.04 |
|  | subsample | 0.8 |
|  | colsample_bytree | 0.8 |
|  | min_child_weight | 7 |
|  | gamma | 0.2 |
|  | reg_alpha | 1.0 |
|  | reg_lambda | 3.0 |
| Random forest | n_estimators | 100 |
|  | max_depth | 3 |
|  | min_samples_split | 30 |
|  | min_samples_leaf | 15 |
|  | max_features | sqrt |
|  | max_samples | 0.6 |
|  | ccp_alpha | 0.02 |
| SVM | Kernel | RBF |
|  | C | 0.003 |
|  | gamma | scale |

*Model performance was evaluated using stratified 5-fold cross-validation with AUC as the performance metric. Predicted probabilities from the final XGBoost model were calibrated using the sigmoid method under stratified 5-fold cross-validation.*
