# Supplementary material for: Early prediction of plastic bronchitis in pediatric patients with Mycoplasma pneumoniae pneumonia by interpretable machine learning algorithms
Source: Front Cell Infect Microbiol. 2026 Apr 23;16:1785189. doi: 10.3389/fcimb.2026.1785189 (PMC13149268; doi:10.3389/fcimb.2026.1785189)
Supplement: Supplementary Table 3 — Feature selection with the LASSO algorithm. [file Table3.docx]

**Table S3. The regression coefficients of the variables in the Lasso regression.**

| **Variables** | **Coefficient** |
| --- | --- |
| RBP | -0.155363 |
| Ct value | -0.110301 |
| Duration of fever before admission | 0.088360 |
| Pleural effusion | 0.075631 |
| D-Dimer | 0.045997 |
| Pulmonary consolidation | 0.040952 |
| PLR | -0.040581 |
| Pericardial effusion | -0.039185 |
| LOS | 0.036549 |
| CAR | 0.035643 |
| Lymphocytes | 0.034941 |
| Age | 0.027622 |
| Prodromal symptom duration | 0.019707 |
| PA | 0.019482 |
| Prehospital antibiotics usage | 0.009558 |
| SAA | 0.006263 |
| MRMP | -0.001427 |

Optimal Lambda= 0.013219411484660288

Abbreviations: MRMP: macrolide-resistant *Mycoplasma pneumoniae*; LOS: length of hospital stay; PA: prealbumin; CAR: CRP-to-albumin ratio; SAA: serum amyloid protein A; RBP: retinol-binding protein; PLR: platelet-to-lymphocyte ratio; Ct values: cycle threshold values.
